# Potential local adaptation in populations of invasive reed canary grass (*Phalaris arundinacea*) across an urbanization gradient

**DOI:** 10.1002/ece3.7938

**Published:** 2021-07-28

**Authors:** Leah M. Weston, Kali Z. Mattingly, Charles T. C. Day, Stephen M. Hovick

**Affiliations:** ^1^ Department of Evolution, Ecology and Organismal Biology The Ohio State University Columbus OH USA; ^2^ Plant Pathology and Plant‐Microbe Section School of Integrative Plant Science Cornell University Geneva NY USA

**Keywords:** adaptive plasticity, root mass ratio, root traits, stress tolerance, urban evolution, wetland invader

## Abstract

Urban stressors represent strong selective gradients that can elicit evolutionary change, especially in non‐native species that may harbor substantial within‐population variability. To test whether urban stressors drive phenotypic differentiation and influence local adaptation, we compared stress responses of populations of a ubiquitous invader, reed canary grass (*Phalaris arundinacea*). Specifically, we quantified responses to salt, copper, and zinc additions by reed canary grass collected from four populations spanning an urbanization gradient (natural, rural, moderate urban, and intense urban). We measured ten phenotypic traits and trait plasticities, because reed canary grass is known to be highly plastic and because plasticity may enhance invasion success. We tested the following hypotheses: (a) Source populations vary systematically in their stress response, with the intense urban population least sensitive and the natural population most sensitive, and (b) plastic responses are adaptive under stressful conditions. We found clear trait variation among populations, with the greatest divergence in traits and trait plasticities between the natural and intense urban populations. The intense urban population showed stress tolerator characteristics for resource acquisition traits including leaf dry matter content and specific root length. Trait plasticity varied among populations for over half the traits measured, highlighting that plasticity differences were as common as trait differences. Plasticity in root mass ratio and specific root length were adaptive in some contexts, suggesting that natural selection by anthropogenic stressors may have contributed to root trait differences. Reed canary grass populations in highly urbanized wetlands may therefore be evolving enhanced tolerance to urban stressors, suggesting a mechanism by which invasive species may proliferate across urban wetland systems generally.

## Graphical abstract

We tested whether populations of a ubiquitous wetland invader, reed canary grass (*Phalaris arundinacea*), differed phenotypically across an urbanization gradient, suggesting evolutionary responses to common urban stressors. We found clear trait and trait plasticity variation among populations, suggesting reed canary grass may be evolving enhanced tolerance to anthropogenic stressors.

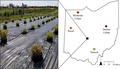

## 1 INTRODUCTION

Humans dramatically alter their local environments. Compared to systems less intensively impacted, human‐dominated environments experience shifts such as higher air temperatures (Li et al., 2011), altered hydrology and soil structure (Poor & McDonnell, 2007; Pouyat et al., 1995), and contamination with metals and road salt (Cunningham et al., 2008; Kumar & Hundal, 2016). Humans also shape their biotic environments, favoring some species, either intentionally or unintentionally, that can tolerate these novel selective pressures and disfavoring others. Recent studies have begun to provide evidence that urban selective regimes are important drivers of natural selection (Borden & Flory, 2021; Lambrecht et al., 2016; Yakub & Tiffin, 2017). As global human impacts accelerate, it is increasingly important to understand how anthropogenic forces shape species evolution.

In the face of novel anthropogenic selective pressures, introduced and invasive species are particularly well‐positioned to benefit. High relative abundances of introduced species have become a common feature of many anthropogenically altered systems (King & Hovick, 2020), with these populations often representing introductions from multiple source populations (Dlugosch & Parker, 2008). Adaptive genetic diversity in introduced populations may thus exceed that in native range populations (Dlugosch et al., 2015), priming these species for potential responses to novel selective pressures and thus local adaptation in the introduced range (Dlugosch & Parker, 2008; Hodgins et al., 2018). In fact, introduced species now provide some of our most compelling examples of contemporary evolution (Borden & Flory, 2021; Colautti & Lau, 2015).

For introduced and native species alike, an important component of stress response and local adaptation is thought to be phenotypic plasticity (Chapin et al., 1993; Rivera et al., 2021), or how a phenotype changes in response to environmental conditions. Both individual trait and trait plasticity values can be adaptive (associated with fitness benefits). Although plasticity can buffer selection and thus limit local adaptation in certain circumstances (Schlichting, 2004), it can also be an important component of adaptation (Kelly, 2019), particularly in environmentally heterogenous conditions (Palacio‐López et al., 2015) and across stress gradients (Chevin & Hoffmann, 2017). Plasticity is more likely to be adaptive in spatially or temporally variable environments, as shown in simulations (Berrigan & Scheiner, 2004; Wang et al., 2017) and empirical studies of taxa inhabiting variable urban landscapes (Brans et al., 2017; Esperon‐Rodriguez et al., 2020; Miranda, 2017). Many invasions are also characterized by high and adaptive plasticity (Davidson et al., 2011), implicating plasticity as an important contributor to evolution in introduced species; we suggest this may be especially likely in highly human‐impacted systems due to the temporal variability with which stressors move across such landscapes (Poor & McDonnell, 2007).

Reed canary grass (*Phalaris arundinacea*) is a pervasive wetland invader with high phenotypic and genetic diversity in the introduced range (Lavergne & Molofsky, 2007). Substantial genetic diversity within introduced populations of reed canary grass has been documented (Anderson et al., 2016; Gifford et al., 2002; Nelson et al., 2014), but the extent to which reed canary grass varies in traits among populations in the introduced range is not clear. In addition, no studies have examined population‐level variability in response to relevant anthropogenic selective forces, which is crucial to understanding how local adaptation may have driven this species' success as an invasive species.

Previous research suggests that some genotypes of reed canary grass are highly plastic across stress gradients (Martina & von Ende, 2012) and vary in tolerance to common urban wetland contaminants like salt and copper (Haiminen et al., 2014; Marchand et al., 2014; Polechońska & Klink, 2014). Several studies have evaluated reed canary grass performance in wetlands contaminated with copper and zinc (Bernard & Lauve, 1995; Korzeniowska & Stanis, 2011; Korzeniowska & Stanislawska‐Glubiak, 2017; Marchand et al., 2014). The stress response of reed canary grass genotypes to these metals varies substantially, suggesting that certain genotypes may also have superior survival in polluted wetlands. In addition to metals, salt is a pervasive urban wetland contaminant. Although reed canary grass is not known to be particularly salt‐tolerant, previous work with the species has found differential gene expression associated with salt‐tolerant cultivars (Haiminen et al., 2014) and phenotypic diversity under salt stress (Maeda et al., 2006).

To understand the potential for phenotypic plasticity to spur local adaptation of introduced species in response to urban stressors, we used reed canary grass as a model system to ask two complementary questions. First, do populations from wetlands surrounded by different levels of urbanization vary systematically in their response to three common urban contaminants (salt, copper, and zinc)? We expected that site‐specific differences in exposure to and selection by common anthropogenic stressors would mean that reed canary grass populations surrounded by intense urbanization would show fewer negative effects of stressors than reed canary grass surrounded by natural vegetation, with intermediate responses from populations not at those extremes of urbanization level. Second, we asked: Do populations differ in phenotypic plasticity, and is plasticity associated with fitness? Given the link between plasticity and habitat variability in urban spaces, we expected plants from urban populations to be more plastic in response to stressors than plants from populations adjacent to other land uses. Additionally, we expected plasticity to be positively associated with fitness (i.e., adaptive) in this system, as high plasticity is commonly associated with invasion success.

## 2 METHODS

### 2.1 Source population selection and sampling

**FIGURE 1:**
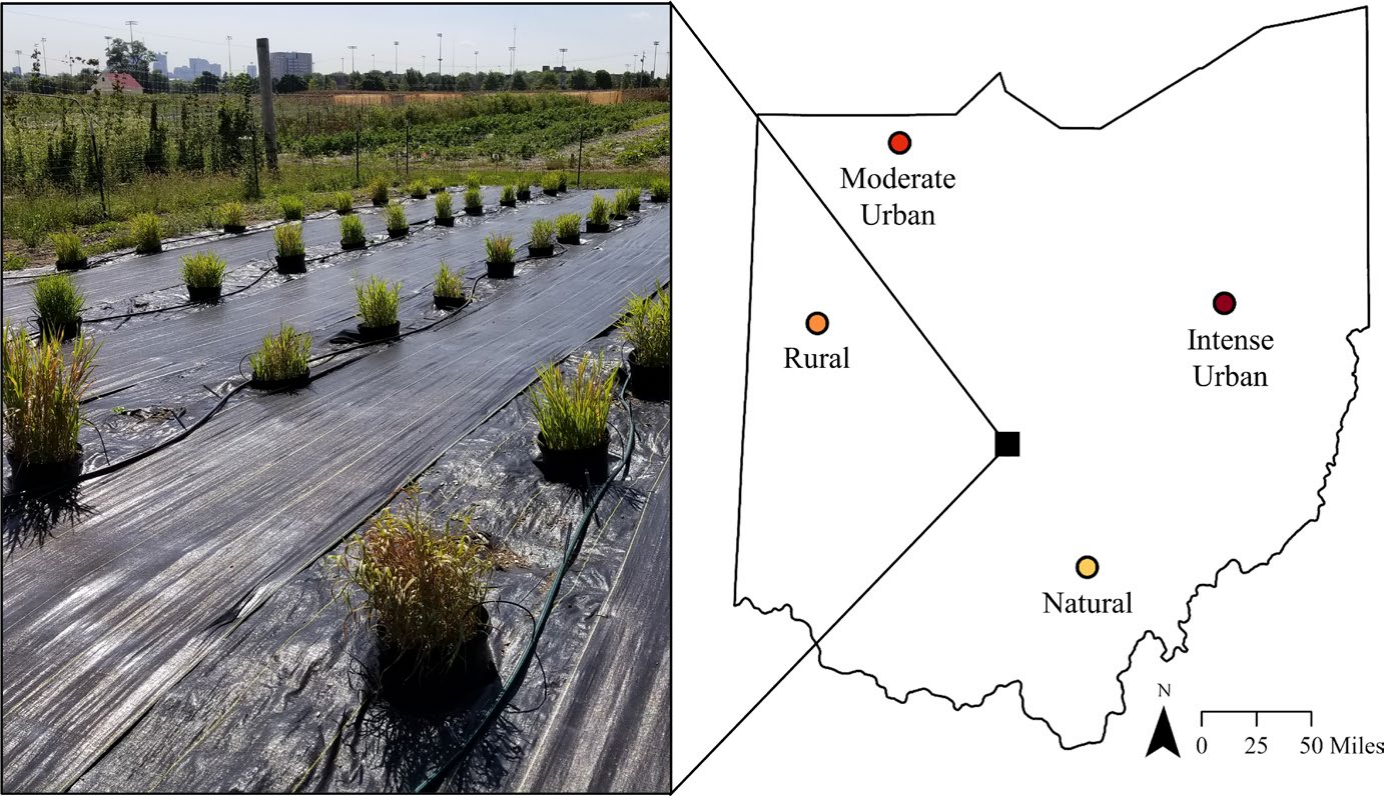
Site map of the four source populations and location of the common garden experiment. Reed canary grass (*Phalaris arundinacea*) seeds from four source populations were collected from wetlands in Ohio, USA, and grown together in a common garden experiment in Columbus, Ohio. The picture illustrates reed canary grass growing in flooded pots during July 2018. In the key, Nat. = natural population, Rur. = ural population, Mod. = moderate urban population, Int. = intense urban population

We collected reed canary grass seeds in July 2017 from four wetlands in Ohio, USA, that varied in predominant land use within a surrounding 30‐m buffer according to the 2011 National Land Cover Database (NLCD) (Figure 1). Our collection sites represent a range and diversity of anthropogenic influences to which reed canary grass may have experienced unique selection pressures; we refer to them here as our natural, rural, moderate urban, and intense urban wetlands. Briefly, these wetlands were required to have ≥50% of buffer area containing the following land‐use types: natural surrounded by forest and pasture, rural surrounded by pasture and crops, moderate urban surrounded by open and low‐intensity development, and intense urban surrounded by medium‐ and high‐intensity development. Detailed site selection criteria are described in King and Hovick (2020).

We collected six mineral soil cores (15 cm deep × 2 cm diameter) from each site and combined them at the site level to create a composite sample for analysis of metal and micronutrient concentrations via nitric acid digestion (conducted by The Ohio State University's Service Testing and Research Laboratory). Metal and micronutrient information from the composite soil sample provides a snapshot of contaminant levels at one timepoint but does not provide information about temporal or spatial variability of contaminants (Carter & Gregorich, 2008). We present site‐level data in Table 1 to further contextualize similarities and differences among sites beyond our a priori site selection criteria, including percent impervious surface and the land‐use categories that account for ≥70% of the area within a larger, 250‐m buffer surrounding our wetlands. This larger buffer is used to better illustrate conditions at a scale that is likely to have exerted an influence on our focal populations. Impervious surface and land‐use data were based on the 2011 ArcMap Imagery Base Map and the National Land Cover Database after experiment completion.

**TABLE 1:** Site information from four source population wetlands where reed canary grass seeds were collected

Source population	Site latitude	Site longitude	Soil Na (ppm)	Soil Cu (ppm)	Soil Zn (ppm)	Impervious surface within 250‐m buffer (%)	Area in predominant land‐use categories within 250‐m buffer (%)
Natural	39.3629	−82.4397	130.2	23.42	71.69	2.3	63.6% Deciduous Forest 24.1% Pasture/Hay
Rural	40.6102	−84.3314	129.8	26.56	97.28	1.5	63.2% Cultivated Crops 30.9% Pasture/Hay
Moderate Urban	41.5755	−83.8021	52.46	11.35	19.52	4.1	26.9% Developed, Open Space 26.7% Cultivated Crops 19.8% Deciduous Forest
Intense Urban	40.7637	−81.5213	131.4	101.6	596.6	45.5	45.7% Developed, High Intensity 34.7% Cultivated Crops

Note

Wetlands were chosen based on land‐use categories within a 30‐m buffer, but impervious surface and land‐use categories accounting for ≥70% of the area within a 250‐m buffer are shown here to better illustrate conditions at a scale that likely influenced our focal populations. Impervious surface and land‐use data were determined after experiment completion, based on the 2011 ArcMap Imagery Base Map and the National Land Cover Database. Soil micronutrient and metal concentrations were obtained from a one‐time bulk soil analysis (see Section 2).

All seeds were stored at 1.6℃ until 6 April 2018, when they were sown into 50‐cell flats of Sunshine Redi‐Earth Plug & Seedling Mix (Sun Gro Horticulture, Agawam, MA, USA). We grew plants in the greenhouse under a 12‐hr photoperiod at temperatures of 28℃ day/25℃ night, watering as needed and fertilizing weekly with a 150 mg·L − 1 N nutrient solution (21 N‐2.2 P‐16.6 K Jack's All‐Purpose Liquid Feed, J.R. Peter's Inc., Allentown, PA, USA) after emergence of the second leaf. After 5 weeks, we selected eight large individuals from each population and split each into a set of four genetically identical plants (clones), trimming shoots to 10 cm length to standardize initial size, and abate transplant stress.

After 2 weeks, we randomly selected five replicate four‐clone sets per population for the experiment (*n* = 80 plants total: four clones × five distinct individuals × four populations). We trimmed all plants again to standardize shoot length to 10 cm and root length to 3 cm. We transplanted seedlings into 7.6‐L nursery pots with a 1:1 (v:v) mixture of sand (silica 20/30 grade) and Fafard Mix #2 (Sun Gro Horticulture, Agawam, MA, USA) and then let plants recover from transplant stress in a shaded greenhouse for 5 days before moving them to the field.

### 2.2 Experimental design

On May 30, we moved all pots to a common garden experiment at The Ohio State University's Waterman Agricultural and Natural Resources Laboratory in Columbus, Ohio, USA (40.009833°N, 83.039694°W) (Figure 1). Reed canary grass is a facultative wetland species (Lichvar et al., 2016), so we constructed individual artificial “wetlands” by nesting each 7.6‐L pot into an 18.9‐L pot that had drainage holes only at a point 5 cm below the fill line, letting the soil remain flooded while still permitting overflow drainage. Pots were spaced 1.5 m apart and dug 18 cm into the ground to stabilize them and insulate the root zone from solar radiation. We irrigated with drip emitters as needed to maintain at least 5 cm of standing water in the bottom of the larger pot. The plot was surrounded with 1.2 m tall fencing to reduce mammalian herbivory.

We used a randomized block design, with each block containing one set of clones from each population and five blocks in total. The four clones from each population were randomly selected to receive one of four treatments: control (no stressor), salt stress, zinc stress, or copper stress. All plants were also fertilized with Hoagland's nutrient solution (Hoagland & Arnon, 1950). We increased the concentrations and application rate of our treatments as plants grew larger and thus transpired more; when we did so, stressor and fertilizer concentrations were increased in proportion with one another to minimize the likelihood that nutrient limitation instead of our focal stressors led to performance losses. The initial concentrations of our stressor solutions were 0.472 g sodium (NaCl) per 100 ml solution, 5 mg zinc (ZnSO_4_ * 7H_2_O) per 100 ml solution, and 1 mg copper (CuSO_4_ * 5H_2_O) per 100 ml solution. We applied nutrients and treatments once weekly until week six and then twice weekly through week eight, at which point we maintained a twice weekly application rate and doubled all concentrations to 2× Hoagland's nutrient solution plus either 0.944 g sodium, 10 mg zinc, or 2 mg copper per 100 ml solution. In week 10, we doubled concentrations again to 4× Hoagland's nutrient solution plus either 1.888 g sodium, 20 mg zinc, or 4 mg copper per 100 ml solution. The common garden experiment was maintained for 18 weeks before destructive sampling. Over the course of the experiment, control pots received 9.84 mg Na, 0.164 mg Cu, and 0.41 mg Zn from 3 L of Hoagland's solution. Plants in the salt treatment received an additional 38.7 g Na, in the copper treatment an additional 82 mg Cu, and in the zinc treatment an additional 410 mg Zn from a cumulative 3 L of treatment solutions.

### 2.3 Plant trait measurements

At the end of the growing season, we collected data necessary to quantify 10 functional traits that we expected would be relevant for reed canary grass performance (see Table 2). From aboveground tissues, we recorded dry aboveground biomass, chlorophyll content, height, leaf area, leaf fresh weight, and leaf dry weight. For leaf measurements, we used the newest fully expanded leaves. We harvested three leaves per pot by cutting them at the junction of the leaf blade and leaf sheath. We recorded fresh weight, imaged leaves with an Epson Perfection V800 scanner (Epson America Inc., San Jose, CA, USA), and analyzed images using WinFolia Pro 2015a software (Regent Instruments Inc., Québec, Canada). Then, we estimated chlorophyll content using an Apogee MC10 meter (Apogee Instruments Inc., Logan, Utah, USA) by averaging 3 measurements taken at the midrib of the leaf blade. After scanning, leaves were oven‐dried at 65℃ for 48 hr and then weighed. We calculated specific leaf area as the ratio of leaf area to leaf dry mass and leaf dry matter content as the ratio of leaf dry weight to leaf fresh weight (Pérez‐Harguindeguy et al., 2013).

**TABLE 2:** Means (and standard errors) for PC axis scores and traits by treatment and source population

	Treatment effects from two‐way ANOVA						Source population effects from two‐way ANOVA					
	*F*	*p*	Control	Salt	Copper	Zinc	*F*	*p*	Natural	Rural	Moderate	Intense
PC axis 1	**47.01**	**<.001**	0.76 (0.09)	−0.46 (0.09)***	−0.05 (0.08)***	−0.24 (0.08)***	**6.145**	**<.001**	−0.17 (0.14)^b^	−0.12 (0.13)^b^	0.03 (0.14)^ab^	0.26 (0.12)^a^
PC axis 2	**6.669**	**<.001**	0.26 (0.14)	−0.34 (0.11)***	−0.12 (0.11)*	0.21 (0.14)	**4.312**	.**008**	0.31 (0.13)^a^	0.00 (0.15)^ab^	−0.07 (0.13)^ab^	−0.24 (0.10)^b^
PC axis 3	2.070	.112	0.26 (0.12)	−0.04 (0.14)	−0.19 (0.11)	−0.03 (0.15)	1.441	.238	0.18 (0.17)	0.00 (0.12)	−0.20 (0.11)	0.02 (0.11)
Aboveground Biomass (g)	**66.66**	**<.001**	63.8 (3.0)	27.0 (1.5)***	41.2 (1.9)***	37.4 (1.7)***	**6.510**	**<.001**	42.0 (3.5)^b^	39.2 (2.9)^b^	38.8 (4.3)^b^	49.3 (3.6)^a^
Height (cm)	**7.590**	**<.001**	54.7 (1.4)	46.9 (1.2)***	50.4 (1.1)**	49.6 (1.1)**	2.543	.063	50.2 (1.2)	48.7 (1.5)	49.7 (1.3)	53.1 (1.2)
Chlorophyll Content (µmol/m^2^)	**104.1**	**<.001**	12.8 (0.6)	5.5 (0.3)***	5.8 (0.2)***	5.9 (0.3)***	1.277	.292	7.8 (0.7)	7.0 (0.7)	7.7 (0.8)	7.5 (0.9)
Leaf Dry Matter Content (g_dry_/g_fresh_)	1.857	.145	0.34 (0.00)	0.35 (0.01)	0.36 (0.01)	0.35 (0.01)	**3.487**	.**020**	0.34 (0.01)^b^	0.35 (0.01)^ab^	0.36 (0.01)^a^	0.36 (0.01)^a^
Specific Leaf Area (cm^2^/g)	**3.114**	.**032**	0.23 (0.00)	0.24 (0.01)	0.24 (0.01)	0.25 (0.01)**	2.728	.051	0.25 (0.01)	0.24 (0.01)	0.23 (0.01)	0.23 (0.01)
Belowground Biomass (g)	**12.74**	**<.001**	198 (21)	82.2 (6.0)***	143 (11)*	180 (17)	2.023	.119	178 (21)	144 (16)	149 (21)	130 (8.6)
Root Mass Ratio (g_root_/g_total_)	**6.204**	**<.001**	0.74 (0.02)	0.75 (0.01)	0.77 (0.01)	0.81 (0.02)***	**5.474**	**<.001**	0.79 (0.01)^a^	0.77 (0.02)^ab^	0.78 (0.01)^a^	0.72 (0.01)^b^
Specific Root Length (cm/mg)	**4.190**	.**009**	0.30 (0.01)	0.41 (0.03)**	0.33 (0.03)	0.36 (0.02)	**7.891**	.**001**	0.45 (0.04)^a^	0.32 (0.02)^b^	0.31 (0.02)^b^	0.32 (0.02)^b^
Fine Root Diameter (mm)	0.468	.706	0.50 (0.02)	0.49 (0.02)	0.50 (0.01)	0.47 (0.02)	1.154	.334	0.47 (0.02)	0.49 (0.01)	0.51 (0.01)	0.50 (0.01)
Fine Root Length (mm)	1.310	.278	76.0 (3.9)	69.6 (2.7)	67.9 (2.8)	72.9 (3.7)	2.469	.069	65.9 (3.2)	77.7 (3.3)	71.7 (3.2)	71.5 (3.3)

Note

General linear models used treatment and source population as predictors of PC axis scores and trait values. Degrees of freedom (*df*) = 3,69 for tests of treatment and source population effects. Dunnett's tests were used to test for differences between individual stress treatments versus control: * = *p* < .05, ** = *p* < .01, *** = *p* < .001. Superscript letters represent significant Tukey differences among source populations at *p* < .05.

From belowground tissues, we recorded dry belowground biomass and fine root length, diameter, and dry mass. After harvesting aboveground biomass, we kept belowground biomass in pots at 4℃ until root washing. Roots were washed by repeatedly rinsing them over a series of three sieves (mesh sizes of 3.35, 2.0, and 0.5 mm) to capture fine roots until all growing media had been removed. Samples were oven‐dried at 65℃ for at least 72 hr and weighed. Root mass ratio was calculated as the ratio of dry root biomass to total dry aboveground and belowground biomass. To determine root tissue concentrations of Na, Cu, and Zn after experiment completion, approximately 2 g dried root tissue was ground from a subset of all plants (*n* = 59 of 80 because some samples were misplaced after root trait measurement completion) and analyzed via nitric acid microwave digestion (conducted by The Ohio State University's Service Testing and Research Laboratory).

During the root washing process, we separated ten fine roots from the bulk root mass of each pot to measure fine root length and fine root diameter. These samples were unbroken roots <2 mm in diameter that could be traced from root tip to a rhizome attachment point. We stained fine roots by soaking them in a methylene blue solution (5 g/L) for 5 min to enhance contrast for imaging (Roumet et al., 2008). After staining, fine roots were spread out in a single layer in a 1.5 cm deep transparent tray (22 cm × 25 cm) that was filled with water and scanned. Fine root samples were then oven‐dried at 65℃ and weighed. Fine root length and diameter were measured using WinRHIZO Pro 2019a software (Regent Instruments, Quebec, Canada). To calculate pot‐level metrics for fine root length, diameter, and dry mass, we took the mean across ten fine root samples per pot. We calculated specific root length by dividing mean dry fine root biomass by mean fine root length (Pérez‐Harguindeguy et al., 2013).

### 2.4 Statistical analysis

All analyses were conducted using R version 3.5.1 (R Core Team, 2018). We performed principal component analysis (PCA) using the R package vegan (Oksanen et al., 2018) to reduce dimensionality in our dataset of ten traits collected from all plants and to visualize potential differences across treatments and source populations in suites of traits. Trait values were centered and standardized prior to performing PCA. The most highly correlated traits were aboveground biomass and chlorophyll content (*r* = 0.765); all other correlation coefficients were less than 0.67 (Table A1).

We tested the effects of treatment and source population on multitrait variation using PCA scores, as well as on individual traits and trait plasticities. For each trait, we quantified plasticity in response to a given stressor by subtracting the trait value of a control plant from the trait value of the genetically identical plant exposed to stress and then dividing by the trait value of the control plant (a modified phenotypic plasticity index; Valladares et al., 2006). To test for treatment and source population differences, we used the R package nlme (Pinheiro et al., 2018) to conduct ANOVA using mixed effects models fit with maximum likelihood. Models included treatment and population as fixed effects and block as a random effect. In all models, we tested the interaction between treatment and source population but found no support for it (based on statistical significance of the interaction, AIC values, and likelihood ratio tests), so we present results only from the simpler, no‐interaction models. In the Results text, reported *p*‐values are from these simpler models unless otherwise specified. In most cases, we used Tukey corrections after finding significant effects from the ANOVA model to make post hoc inferences about pairwise differences using the R package multcomp (Torsten et al., 2008). We discuss results from significant post hoc comparisons in cases where the overall effect of source population was either significant (*p* < .05) or marginally so (0.05 < *p* < .1) because of our a priori interest in testing the hypothesis of variation among populations. When PCA scores or mean trait values varied significantly by treatment, we used Dunnett's corrections to assess whether individual stressor treatments differed from control (Bretz et al., 2011). For the main effects of treatment and source population, we report effect sizes as percent change in a given trait or its plasticity, averaging the effect of treatment across populations and averaging the effect of population across treatments.

Lastly, we used mixed effects models to test whether plasticity in each trait was adaptive (i.e., positively associated with fitness) under stressful conditions, using total biomass in a given stressor treatment as our fitness proxy. Because plants in our experiment did not flower, we cannot assess adaptive value using fecundity to estimate fitness; however, for clonally reproducing perennials like reed canary grass, biomass is a reasonable alternative (Younginger et al., 2017). Separate adaptive plasticity analyses were conducted for each treatment and trait; thus, all models were constructed to predict total biomass under a given stress treatment in response to variation in plasticity for a given trait and source population (including the trait value itself in the stressed plant as a covariate and block as a random effect, fit via maximum likelihood). As in the models described above, we tested whether the relationship between plasticity and biomass varied by source population, testing the plasticity × population interaction and dropping it where its inclusion was not supported.

## 3 RESULTS

### 3.1 Treatment effectiveness

Our stressor treatments yielded salt, copper, and zinc concentrations in reed canary grass root tissues that far exceeded those in controls. Sample sizes are included because treatment effectiveness was tested on root tissue from a subset of all plants (*n* = 59). Relative to controls (*n* = 16), salt‐treated plants (*n* = 18) had sodium concentrations 5.7 times higher (4.580 ± 0.458 vs. 0.806 ± 0.061 mg/g), copper‐treated plants (*n* = 12) had copper concentrations 4.6 times higher (0.018 ± 0.004 vs. 0.004 ± 0.0003 mg/g), and zinc‐treated plants (*n* = 13) had zinc concentrations 6.0 times higher (0.221 ± 0.059 vs. 0.037 ± 0.002 mg/g).

### 3.2 Variation in multitrait space

**FIGURE 2:**
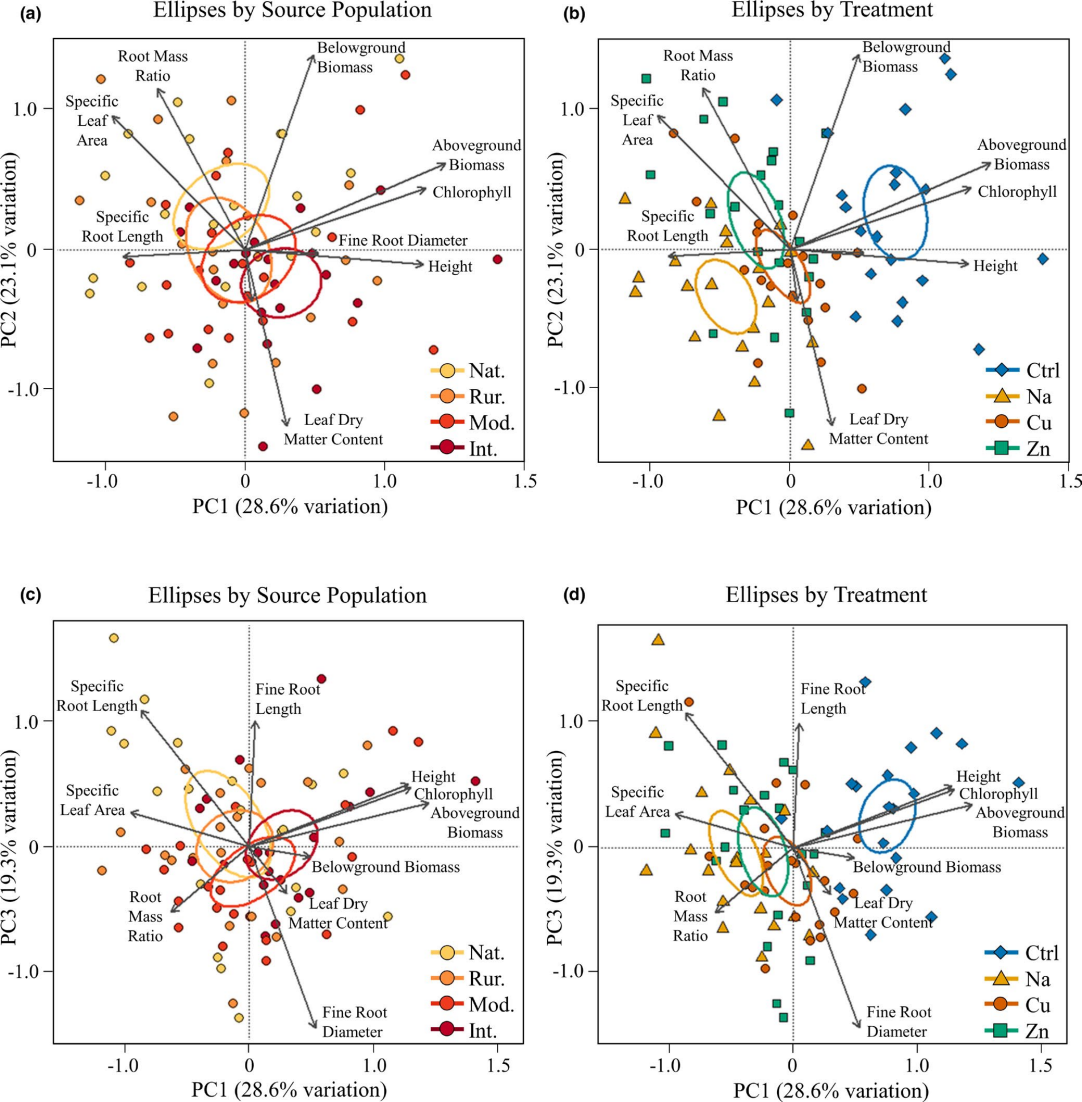
Principal component analysis (PCA) reducing 10 traits to three descriptive axes. The natural and intense urban source populations differed along PC1 and PC2, representing trait differences in size above and belowground and differences in leaf traits (see Table 2). Panels a and b show PC1 versus PC2; panels c and d show PC1 versus PC3. Ellipses indicate 95% confidence intervals by source population (panels a and c) or by stressor treatment (panels b and d). In the source population key, Nat. = natural population, Rur. = rural population, Mod. = moderate urban population, and Int. = intense urban population. In the treatment key, Ctrl. = control, Na = salt addition, Cu = copper addition, Zn = zinc addition

Our principal component analysis (PCA) reduced most of the variation among our ten traits to three axes, representing three key dimensions of trait variability (Figure 2, Table A2). PC Axis 1 (PC1) largely reflected variation in aboveground plant growth and was most highly correlated with aboveground biomass, height, and chlorophyll content. PC Axis 2 (PC2) reflected variability in resource acquisition traits including leaf dry matter content and specific leaf area, as well as in root growth, represented by belowground biomass and root mass ratio. High PC2 scores represent leaf trait values associated with competitiveness (larger, thinner leaves) but root trait values associated with resource conservation (more belowground biomass). PC Axis 3 (PC3) reflected variability in fine root resource acquisition and production (specific root length, fine root diameter, and fine root length), with high values indicating high resource uptake efficiency (longer, thinner fine roots).

PC1 and PC2 scores varied by source population and stress treatment. Plants from the intense urban population had larger PC1 scores than those from natural and rural source populations (both Tukey *p* < .003, Figure 2a, Table 2), indicating that intense urban plants were larger regardless of treatment. Intense urban and natural plants also differed along PC2, indicating that across treatments, increasing urbanization was associated with leaf traits reflecting a resource conservation strategy and reduced belowground biomass (Tukey *p* = .001, Figure 2a, Table 2). All stress treatments had lower mean PC1 scores relative to control (all Dunnett's *p* < .001), and the salt and copper treatments had significantly lower PC2 scores than control (Dunnett's *p* < .001 and 0.0262, respectively; Figure 2b; Table 2), indicating that stressed plants were smaller and expressed leaf traits consistent with a resource conservation strategy. PC3 scores did not vary by treatment or source population (Figure 2c,d, Table 2).

### 3.3 Aboveground trait variation by treatment and population

Aboveground biomass in reed canary grass varied both by stress treatment and source population. All stress treatments reduced aboveground biomass relative to controls (Table 2), with reductions of 57.7% from salt, 35.4% from copper, and 41.3% from zinc treatments (all Dunnett's *p* < .001). Plants from the intense urban population produced 15%–21% more aboveground biomass than those from the other populations, which did not differ from one another (*p* < .001, Table 2). Plasticity in aboveground biomass also differed by treatment and source population (*p* < .001 and *p* = .048, respectively; Table 3), with plants in the salt treatment reducing biomass by 30%–39% more than those in the zinc and copper treatments, compared to control (both Tukey *p* < .001). Plants from the intense urban population were 33% less plastic (i.e., maintained more biomass) under stressful conditions than plants from the moderate urban population, relative to controls (Tukey *p* = .018).

**TABLE 3:** Plasticity means (and standard errors) for each trait by treatment and source population

Traits	Plasticity effects by Treatment from two‐way ANOVA					Plasticity effects by Source Population from two‐way ANOVA					
	*F*	*p*	Salt	Copper	Zinc	*F*	*p*	Natural	Rural	Moderate	Intense
Aboveground Biomass (g)	**17.46**	**<.001**	−0.557 (0.032)^a^ [−0.796, −0.294]	−0.339 (0.030)^b^ [−0.610, 0.407]	−0.391 (0.036)^b^ [−0.747, −0.113]	**2.832**	.**048**	−0.413 (0.050)^ab^ [−0.721, −0.113]	−0.417 (0.035)^ab^ [−0.680,−0.132]	−0.505 (0.051)^a^ [−0.796, −0.181]	−0.381 (0.038)^b^ [−0.583,−0.168]
Height (cm)	2.224	.119	−0.134 (0.028) [0.321, 0.274]	−0.070 (0.025) [−0.343, 0.157]	−0.086 (0.028) [−0.282, 0.301]	**3.333**	.**027**	−0.039 (0.043)^a^ [−0.220, 0.301]	−0.111 (0.019)^ab^ [−0.231, 0.041]	−0.152 (0.029)^b^ [−0.343, 0.012]	−0.086 (0.026)^ab^ [−0.263, 0.115]
Chlorophyll Content (µmol/m^2^)	0.515	.601	−0.553 (0.031) [−0.769, −0.216]	−0.532 (0.025) [−0.713, −0.329]	−0.517 (0.032) [−0.731, −0.231]	**3.205**	.**031**	−0.467 (0.042)^b^ [−0.681, −0.216]	−0.523 (0.033)^ab^ [−0.765,−0.340]	−0.590 (0.019)^a^ [−0.796, −0.476]	−0.555 (0.031)^ab^ [−0.731,−0.324]
Leaf Dry Matter Content (g_dry_/g_fresh_)	0.732	.486	0.036 (0.028) [−0.098, 0.336]	0.053 (0.015) [−0.027, 0.182]	0.022 (0.018) [−0.099, 0.131]	1.026	.389	0.037 (0.024) [−0.045, 0.311]	0.029 (0.023) [−0.099, 0.171]	0.017 (0.019) [−0.091, 0.135]	0.067 (0.030) [−0.098, 0.336]
Specific Leaf Area (cm^2^/g)	1.740	.186	0.071 (0.028) [−0.185, 0.303]	0.033 (0.019) [−0.173, 0.134]	0.093 (0.028) [−0.136, 0.343]	2.235	.096	0.116 (0.028) [−0.085, 0.343]	0.051 (0.027) [−0.136, 0.203]	0.075 (0.027) [−0.067, 0.246]	0.021 (0.033) [−0.185, 0.278]
Belowground Biomass (g)	**11.59**	**<.001**	−0.507 (0.056)^a^ [−0.839, 0.094]	−0.182 (0.074)^b^ [−0.717, 0.407]	0.029 (0.114)^b^ [−0.842, 1.454]	0.762	.521	−0.356 (0.11) [−0.839, 0.378]	−0.159 (0.100) [−0.730, 0.561]	−0.239 (0.16) [−0.842, 1.454]	−0.158 (0.07) [−0.597, 0.373]
Root Mass Ratio (g_root_/g_total_)	**4.034**	.**024**	0.020 (0.024)^b^ [−0.198, 0.251]	0.046 (0.022)^ab^ [−0.111, 0.284]	0.101 (0.020)^a^ [−0.102, 0.281]	2.718	.054	−0.001 (0.026)^b^ [−0.198, 0.179]	0.070 (0.018)^ab^ [−0.029, 0.225]	0.064 (0.029)^ab^ [−0.102, 0.284]	0.090 (0.029)^a^ [−0.107, 0.281]
Specific Root Length (cm/mg)	**3.980**	.**025**	0.360 (0.084)^a^ [−0.119, 1.515]	0.106 (0.080)^b^ [−0.328, 1.224]	0.179 (0.053)^ab^ [−0.277, 0.635]	**6.698**	**<.001**	0.499 (0.118)^a^ [−0.110, 1.515]	0.171 (0.053)^b^ [−0.233, 0.579]	0.092 (0.057)^b^ [−0.297, 0.395]	0.096 (0.068)^b^ [−0.328, 0.595]
Fine Root Diameter (mm)	0.802	.454	−0.004 (0.032) [−0.296, 0.290]	0.016 (0.035) [−0.240, 0.228]	0.035 (0.034) [−0.276, 0.334]	**4.994**	.**004**	−0.110 (0.033)^a^ [−0.296, 0.190]	0.009 (0.039)^b^ [−0.269, 0.228]	0.006 (0.029)^b^ [0.216, −0.166]	0.065 (0.042)^b^ [−0.276, 0.334]
Fine Root Length (mm)	0.385	.682	−0.493 (0.045) [−0.366, 0.569]	−0.516 (0.074) [−0.471, 0.952]	0.008 (0.069) [−0.459, 0.587]	1.877	.146	−0.900 (0.061) [−0.471, 0.460]	0.063 (0.092) [−0.380, 0.952]	−0.117 (0.053) [−0.441, 0.186]	0.020 (0.077) [−0.459, 0.587]

Note

Negative plasticity values indicate control >treatment trait values, whereas positive values indicate treatment >control. Linear mixed models used treatment and source population as predictors of plasticity in each trait. Degrees of freedom *df* = 2,50 for treatment and *df* = 3,50 for source population. Superscript letters represent significant Tukey differences among treatments or source populations at *p* < .05.

Reed canary grass height was reduced by stressor treatments, and height plasticity varied by source population. All stressor treatments reduced plant height relative to controls (*p* < .01, Table 2), with reductions of 7.9%–14.3%. Source populations did not differ in height (*p* = .063, Table 2), but did differ in height plasticity (*p* = .027, Table 3). The natural population was 3.9 times less plastic (i.e., maintained greater height) in response to stress compared to moderate urban plants (Tukey *p* = .006). Height plasticity did not vary by treatment (*p* = .119, Table 3). One outlier genotype from the natural source population exhibited phenotypic patterns that contrasted with all other genotypes, where stressor‐treated plants were taller than the control plant. We found no evidence of treatment misapplication based on root tissue concentrations of stressors at the end of the experiment (zinc‐treated plant +0.276 mg Zn/g compared to control; copper‐treated plant +0.003 mg Cu/g; data not available for salt‐treated plant). This pattern did not persist for other traits, so this genotype was included in all analyses.

Chlorophyll content was reduced by over half in all stressor treatments (*p* < .001, Table 2), whereas source populations varied only in chlorophyll content plasticity. Compared to controls, salt treatments reduced chlorophyll content by 57.1%, copper reduced it by 54.7%, and zinc reduced it by 53.6% (all Dunnett's *p* < .001). Source populations did not differ in chlorophyll content (Table 2), but chlorophyll content plasticity in plants from the moderate urban population was 20.7% greater than in plants from the natural source population (*p* = .031, Tukey *p* = .009, Table 3), reflecting a greater chlorophyll decrease in response to stress by those plants. Chlorophyll content plasticity was similar across all stressor treatments (*p* = .515, Table 3).

Leaf dry matter content differed by source population, but not by treatment (*p* = .145, Table 2). Leaf dry matter content was lowest in plants from the natural source population, with values 5.6%–6.1% lower than plants from the moderate and intense urban populations (Tukey *p* = .038 and 0.013, respectively). Plasticity in leaf dry matter content did not differ by treatment or source population (*p* = .486 and 0.389, respectively; Table 3).

Specific leaf area increased only with zinc treatment relative to controls (Dunnett's *p* < .01, Table 2) and did not vary by source population (*p* = .051, Table 2). Plasticity in specific leaf area did not vary by treatment or source population (*p* = .186 and 0.096, respectively; Table 3).

### 3.4 Belowground trait variation by treatment and population

Belowground biomass and belowground biomass plasticity varied by treatment (both *p* < .001) but not by source population (*p* = .119 and *p* = .521, respectively; see Tables 2 and 3). Salt reduced belowground biomass by 58.5% (Dunnett's *p* < .001) and copper reduced it by 28.0% (Dunnett's *p* = .012). Zinc did not affect belowground biomass. Zinc and copper‐treated plants were therefore less plastic, maintaining more belowground biomass relative to salt‐treated plants (Tukey *p* < .007).

Root mass ratio and its plasticity varied by treatment (*p* < .001 and 0.024, respectively) as well as by source population, although the population difference in plasticity was marginally significant (*p* = .002 and 0.054, respectively; see Tables 2 and 3). Zinc, the only treatment to affect root mass ratios, increased them by 9.6% (Dunnett's *p* < .001). This proportional increase in root biomass reflects the fact that zinc decreased aboveground biomass production while not affecting belowground biomass. Zinc‐treated plants also experienced plastic root mass ratio increases that were 5.2 times greater than salt‐treated plants (Tukey *p* = .009). Plants from the intense urban population had root mass ratios that were 9.5% and 7.6% smaller than those from the natural and moderate urban populations (Tukey *p* < .001 and *p* = .007), reflecting the fact that intense urban plants tended to produce less biomass belowground but more aboveground than the other three source populations. Marginally significant source population differences in root mass ratio plasticity were driven by greater plasticity values in plants from the intense urban versus the natural population (Tukey *p* = .024). This in turn reflects larger stress‐induced decreases in aboveground relative to belowground biomass in the intense urban source population (plasticity belowground = −0.158 ± 0.07 and aboveground = −0.381 ± 0.038; Table 3), whereas aboveground and belowground biomass decreased to a similar degree in the natural source population (plasticity belowground = −0.356 ± 0.11 compared to aboveground = −0.413 ± 0.05; Table 3).

Specific root length and specific root length plasticity differed by treatment (*p* = .009 and 0.025, respectively) and source population (*p* = .001 and *p* < .001, respectively; Tables 3. 2 and 3). Salt was the only treatment to affect specific root length relative to controls, increasing it by 36.7% (Dunnett's *p* < .001) and resulting in longer fine roots per unit of mass. Specific root length plasticity varied similarly by treatment, with plants in the salt treatment plastically increasing specific root length 3.4 times more than plants in the copper treatment (Tukey *p* = .011). Across treatments, plants from the natural source population had specific root lengths with means of 37.8%–43.5% greater than all other populations (all Tukey *p* < .05). Specific root length plasticity was also 292%–544% greater in the natural source population compared to all other source populations (all Tukey *p* < .05).

Fine root diameter did not differ by treatment (*p* = .706) or source population (*p* = .334, Table 2), but fine root diameter plasticity did vary by population (*p* = .004, Table 3). Plants from the natural population tended to grow thinner fine roots in response to stress, reducing fine root diameter by 11%, whereas all other populations increased it by 1%–6% (all Tukey *p* < .05). Fine root diameter plasticity did not vary by treatment (*p* = .454, Table 3).

Fine root length did not vary by treatment or source population (*p* = .278 and *p* = .069, respectively; Table 2), nor did its plasticity (*p* = .682 and *p* = .146, respectively; Table 3).

### 3.5 Relationship of trait plasticities to total biomass

**FIGURE 3:**
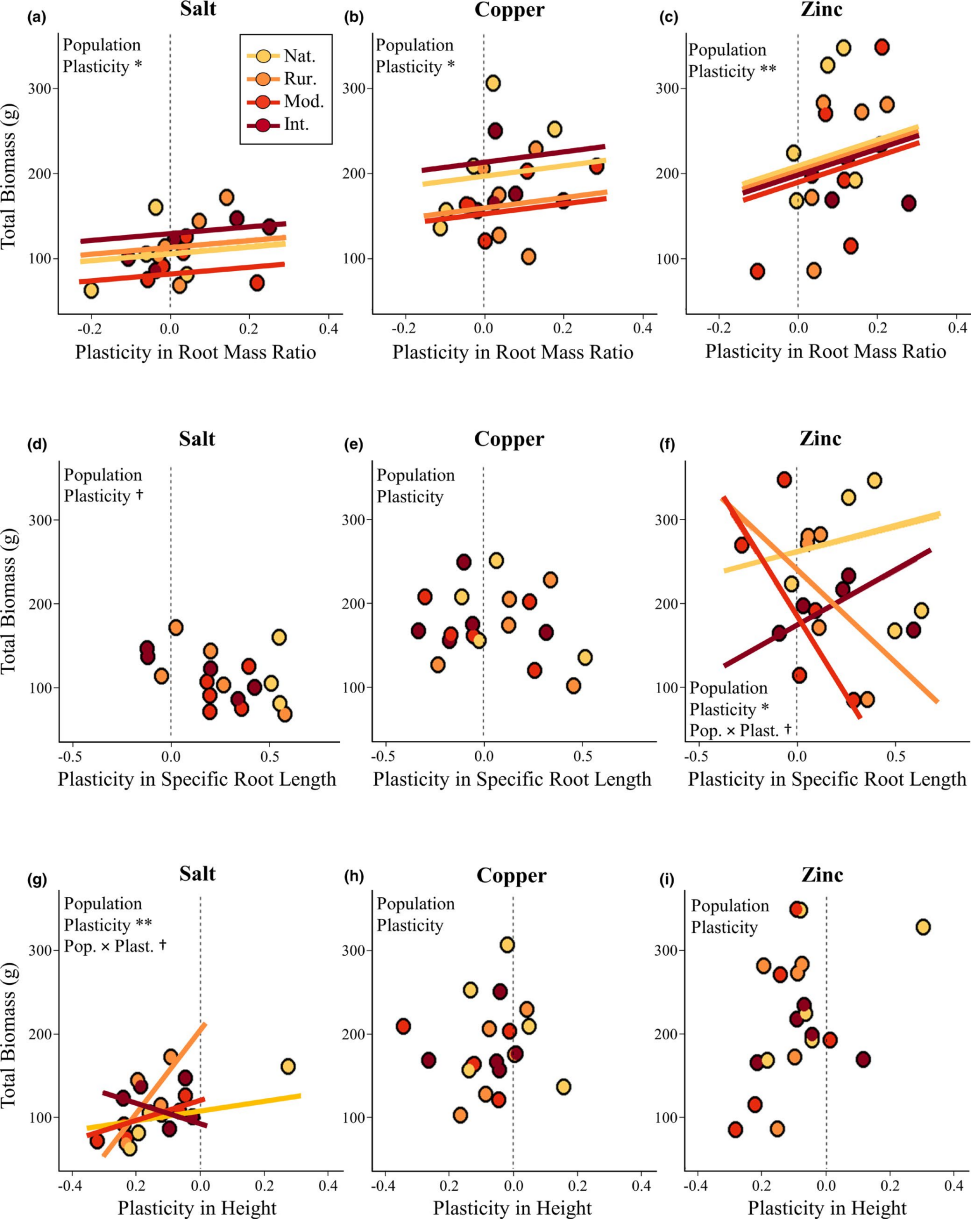
Relationships between total biomass under stressful conditions and plasticity in root mass ratio (a–c), specific root length (d–f), or height (g–i), separated by stressor treatment. Trait plasticity is associated with fitness under at least one stress treatment for each of the traits shown here. A summary of results from each statistical model is shown in the relevant panel; see Table 4 for details. Every model included source population (Population) and trait plasticity (Plasticity). In two cases (panels f and g), the interaction between trait plasticity and source population (Pop. × Plast.) was also included and is marginally significant, indicating the relationship between total biomass and trait plasticity may depend on population. Plasticity values greater than 0 indicate treated plants with trait values that were larger than genetically identical control plants, whereas values less than 0 indicate the opposite. Best‐fit lines represent population‐specific relationships between plasticity and biomass, based on parameter estimates from the final statistical model for each trait‐stressor combination. Significance levels are indicated as: ^†^ = 0.1 > *p* > .05, * = *p* <.05, ** = *p* < .01, *** = *p* < .001. In the key, Nat. = natural population, Rur. = rural population, Mod. = moderate urban population, and Int. = intense urban population

**FIGURE A1:**
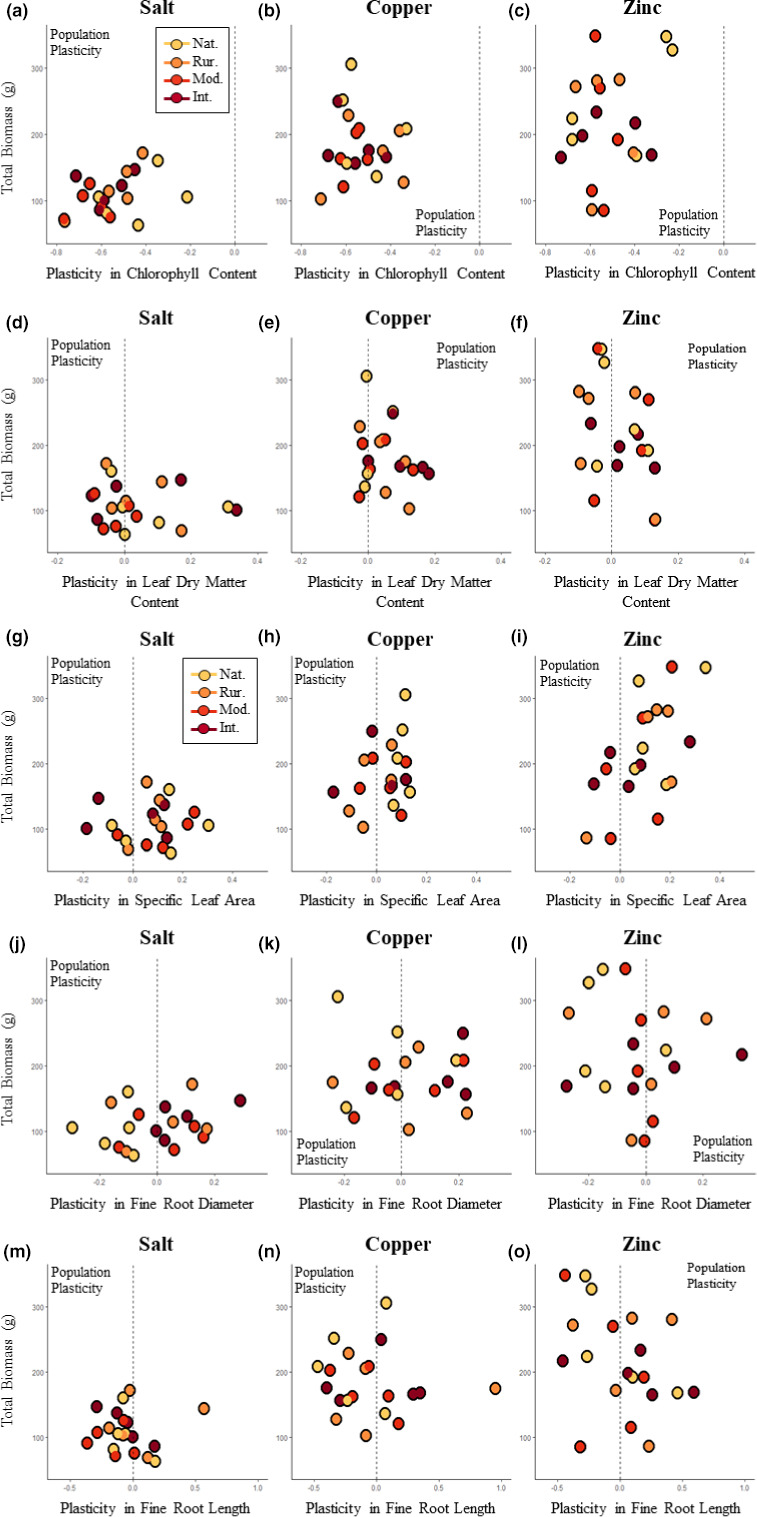
Relationships between total biomass under stressful conditions and plasticity in chlorophyll content (a‐c), leaf dry matter content (d‐f), specific leaf area (g‐i), fine root diameter (j‐l) or fine root length (m‐o), separated by stressor treatment. There was no statistically significant relationship between total biomass and plasticity in these five traits. Plasticity values greater than 0 indicate treated plants with trait values that were larger than genetically identical control plants, whereas values less than 0 indicate the opposite. Summary results from each statistical model are shown in the relevant panel (see Table A3 for details). In the key, Nat. = natural population, Rur. = rural population, Mod. = moderate urban population, and Int. = intense urban population.

We identified five instances of putatively adaptive or maladaptive plasticity, where plasticity was either positively or negatively associated with total biomass, our proxy for fitness: plasticity in root mass ratio in response to all three stressors (all *p* < .05), plasticity in specific root length under zinc stress (*p* = .041) and plasticity in height under salt stress (*p* < .01, Figure 3, Table 3). Plasticities of all remaining traits and across all treatments were not associated with total biomass (all *p* > .05, Figure A1, Table A3).

**TABLE 4:** Statistics from adaptive plasticity models

Traits (Stress Treatment)	Source population		Trait plasticity		Trait value		Source population × Plasticity	
	*F*	*p*	*F*	*p*	*F*	*p*	*F*	*p*
Root Mass Ratio (Salt)	1.59	.254	**6.13**	.**033**	**10.93**	.**008**		
Root Mass Ratio (Copper)	2.29	.141	**8.34**	.**016**	**25.14**	.**001**		
Root Mass Ratio (Zinc)	1.58	.254	**14.20**	.**004**	**27.16**	**<.001**		
Specific Root Length (Salt)	1.00	.431	3.74	.082	1.91	.197		
Specific Root Length (Copper)	0.81	.516	1.03	.334	1.78	.212		
Specific Root Length (Zinc)	1.41	.318	**6.25**	.**041**	0.58	.471	3.97	.061
Height (Salt)	2.65	.130	**21.18**	.**003**	**7.26**	.**031**	3.11	.098
Height (Copper)	0.68	.586	0.01	.921	0.01	.938		
Height (Zinc)	0.67	.589	2.85	.123	0.73	.413		

Note

These models used source population, trait plasticity, and the interaction between them to predict total biomass in the indicated stressor treatment. The trait value itself was also included as a covariate. When the interaction was not supported, it was dropped from the model. *N* = 20 in each case. Numbers in bold represent significance at *p* < .05.

Plastic root mass ratio increases in response to all three stressors were adaptive, indicating that across all source populations and treatments plants that increased root mass ratios when stressed were universally more successful (all *p* < .05, Figure 3a–c, Table 4). Despite this apparent uniformity, plastic changes in the root mass ratio reflected one of three distinct patterns: (a) negative plasticity values reflecting decreased aboveground and belowground biomass, with larger decreases belowground compared to control; (b) positive plasticity values resulting from decreases in aboveground and belowground biomass, but with smaller decreases belowground compared to control; and (c) positive plasticity values resulting from belowground biomass increases compared to control. Source populations did not clearly differ in which of these three patterns were more common (*χ* ^2^ = 8.32, *df* = 6, *p* = .216). However, a comparison between plants from both ends of the urbanization gradient showed that plants from the natural population were more likely to have negative than positive root mass ratio plasticity values (thus tending toward relatively larger reductions in belowground biomass, 9 out of 15 plants) whereas those from the intense urban population were more likely to have positive than negative root mass ratio plasticity values (and tending toward relative maintenance of or increases in belowground biomass, 12 out of 15 plants; *χ* ^2^ = 5.0, *df* = 4, *p* = .025). These results suggest that plastic shifts in root mass ratio tended to be maladaptive in plants from the natural population when stressed but adaptive in those from the intense urban population, despite their similarities in the overall relationship between plasticity and biomass.

Plastic increases in specific root length in response to zinc stress appeared to be either adaptive or maladaptive, depending on the source population (Figure 3f, Table 4). Although the interaction between specific root length plasticity and source population was only marginally significant (*p* = .061), the model with the interaction term clearly fit our data better than a no‐interaction model, based on both AIC (ΔAIC = 7.3) and a likelihood ratio test (*p* = .004). Model parameter estimates indicate that increases in specific root length under zinc stress may have been adaptive in the natural and intense urban source populations but maladaptive in the rural and moderate urban populations. However, analyses conducted with data subsets from individual populations or population groupings did not support the plasticity‐biomass association identified in the full dataset (all *p* > .05).

For plants under salt stress, plasticity in height was maladaptive (*p* < .01, Figure 3g, Table 4). This pattern reflects height decreases in response to stress that accompanied reductions in biomass. Similar to the pattern in specific root length described above, the interaction between height plasticity and source population was marginally significant (*p* = .098) and the interaction model was a better fit than the noninteraction model (ΔAIC = 6.1; likelihood ratio test *p* = .007). Model parameter estimates indicate that height decreases under salt stress are maladaptive for the natural, rural, and moderate source populations. Analyses conducted with data subsets from individual populations did not support the plasticity‐biomass association identified in our full dataset (all *p* > .05).

## 4 DISCUSSION

Our study documents phenotypic divergence among reed canary grass populations collected along an urbanization gradient, with the clearest trait and trait plasticity differences between populations at the ends of that gradient. Compared to the natural population, the intense urban population was larger aboveground and had resource acquisition trait values (leaf dry matter content and specific root length) that indicated greater stress tolerance. These findings suggest that reed canary grass populations in highly urbanized wetlands may be evolving enhanced tolerance of common urban stressors. Trait plasticity also varied by population in over half of the traits measured, indicating that evolutionary changes in plasticity were just as common as changes in the traits themselves. Furthermore, plasticity in root mass ratio and specific root length was adaptive in some contexts, suggesting that natural selection by anthropogenic stressors may have contributed to the phenotypic differences we observed.

### 4.1 Population‐level trait differences

Few studies have documented phenotypic differences occurring among populations of non‐native species, even when those species were introduced many generations previous or from multiple source populations (but see Colautti et al., 2010; Hiatt & Flory, 2020; Matesanz et al., 2012). The usual lack of such observed differences could result from introduced populations tending to harbor substantial variability within populations (Dlugosch & Parker, 2008), which could hinder the detection of among‐population variability. This scenario may often apply to reed canary grass, which is extremely variable within populations (Anderson et al., 2016; Gifford et al., 2002; Nelson et al., 2014) because of multiple presumed introduction events (Lavergne & Molofsky, 2007). However, such within‐population diversity also provides the necessary genetic material for local adaptation, as long as selective landscapes favor different genotypes from place to place. Other research has found reed canary grass genotypes to vary in their response to leaf litter accumulation (Eppinga & Molofsky, 2013) and soil moisture (Nelson & Anderson, 2015), with potential divergence between upland and wetland populations in the latter study.

In our study, population‐level differences in traits and trait plasticities may reflect varying selection pressures across the landscape tied to surrounding land use. Our natural and intense urban source populations differed most noticeably, with the intense urban population having larger PC1 scores (representing greater aboveground biomass, height, and chlorophyll content) and smaller PC2 scores (representing greater leaf dry matter content and belowground biomass, and lower specific leaf area and root mass ratio). The rural population was similar to the natural population in multivariate trait space, while the moderate urban population was intermediate. We highlight three potential drivers behind these patterns, focusing on differences between reed canary grass from natural versus intense urban populations, as these differences could be indicative of natural selection in response to urbanization.

First, increasing anthropogenic land use commonly leads to more nutrient accumulation in wetlands via stormwater runoff (Kaushal et al., 2014). Strong responses to nitrogen enrichment have been documented for reed canary grass (Chen et al., 2017; Kercher & Zedler, 2004; Martina & von Ende, 2012, 2013; Maurer & Zedler, 2002), including increased aboveground biomass production (Chen et al., 2017; Kercher & Zedler, 2004) and decreased proportional allocation to root biomass (Chen et al., 2017; Wetzel & van der Valk, 1998). Although these reports are of plastic phenotypic changes only, selection in nutrient‐enriched urban wetlands likely also favors highly productive individuals across environmental conditions (Keddy et al., 2000). In our study, the intense urban population produced more aboveground biomass than the natural population, which could be the result of selection in nutrient‐rich urban wetlands.

Second, increasing urbanization often leads to greater wetland sedimentation (Houlahan & Findlay, 2004). Reed canary grass is tolerant to burial via sedimentation (Chen et al., 2014, 2017; Pan et al., 2014), and, when combined with nutrient addition, reed canary grass decreases its root mass ratio in response (Chen et al., 2017) In our study, the intense urban population had lower root mass ratio than the natural population, which suggests that evolved changes in response to sedimentation stress could thus also have contributed to the differences we found between populations at the ends of our land‐use gradient.

Third, urban environments are associated with unique environmental stressors (e.g., higher air temperatures [Li et al., 2011], altered hydrology [Poor & McDonnell, 2007], and metal and salt contamination [Kumar & Hundal, 2016; Cunningham et al., 2008]), any of which could select for enhanced stress tolerance. Leaf traits associated with PC2 were congruent with expectations of urban plants growing in a higher stress environment compared to natural plants due to their greater leaf dry matter content. In a study associating intraspecific trait variation in *Arabidopsis thaliana* to Grime's C‐S‐R theory (Grime, 1977), plants with higher leaf dry matter content were more stress‐tolerant than plants with lower leaf dry matter content (May et al., 2017). Our results are thus an indication that urban environments may select for plants that are more able to tolerate stress.

Regardless of the mechanism, there are important implications regarding trait differences between reed canary grass in intense urban versus natural source populations. Adaptation to one urban environment should increase the likelihood of success in other urban systems, as urbanization tends to produce biologically similar environments (McKinney, 2006). Therefore, urban evolved traits may not only help reed canary grass invade neighboring nonurban habitats, but also increase its likelihood of proliferating in urban systems across the landscape (Borden & Flory, 2021). Additionally, urbanized populations of invasive species may be better prepared than native species to tolerate stress from global change (Borden & Flory, 2021). Our study documents trait variability among populations consistent with these expectations, suggesting that further research regarding evolved responses to urbanization is clearly warranted.

Although our source populations varied in both traits and trait plasticity, presumably reflecting evolutionary divergence in response to suites of anthropogenic stressors, we found no evidence for stressor‐specific response variation among populations (i.e., no population × treatment interactions). Thus, our initial hypothesis that populations would differ in response to individual stressors to which they may have differential exposure histories was not supported. This finding may be influenced by the relatively limited power we had to detect population‐level differences due to the small number of source populations in our study. But it could also reflect weak or variable selection by our focal stressors over time or across our land‐use gradient, or substantial gene flow across the landscape. Future studies should include further source populations from different land‐use categories and seek to quantify selection strengths and gene flow. Such approaches would yield valuable insights into putative evolution in response to anthropogenic stressors by reed canary grass.

### 4.2 Population‐level plasticity differences

Characterizing plasticity of invasive species is important because plasticity is thought to contribute to invasions by facilitating success across many environmental conditions. However, meta‐analyses have shown mixed support for the idea that invaders are inherently more plastic than noninvasive or native species (Davidson et al., 2011; Palacio‐López & Gianoli, 2011). Our study is unique, not for finding that plasticity in reed canary grass was common but that it varied by source population. These population‐level distinctions were as pronounced as differences in trait means, reflecting phenotypic flexibility with implications for resource uptake and productivity both above and below ground. Others have discussed intraspecific differences in plasticity between native and introduced populations of invasive species (e.g., Lavergne & Molofsky, 2007; Zou et al., 2009), but plasticity differentiation among introduced populations is rarely explored.

Perhaps even more relevant than documenting plasticity variation is determining whether and when that plasticity may be adaptive. In plants, their trait values contribute strongly to adaptation, but the contributions of phenotypic plasticity are nearly as common (Palacio‐López et al., 2015), though less often characterized. Yet, despite the fact that much of the literature on plasticity in invasive species assumes that plasticity is linked to fitness benefits, plasticity can also be maladaptive if it buffers against selection or represents a shift away from selectively advantageous trait values. Our study shows a clear example of the latter case, with large plastic height responses to salt stress that were maladaptive: Salt‐stressed plants were simply shorter and produced less biomass. Plastic responses to stress may also represent homeostatic rather than fitness‐related shifts that would thus tend to be adaptively neutral (Dudley, 2004). Most of the plastic responses we observed were neutral with respect to biomass, consistent with previous work showing that adaptive plasticity is relatively rare (Auld et al., 2010; Hendry, 2016). However, such adaptive neutrality might also depend on the specific contexts in which we assessed the importance of plasticity. Theory predicts that plasticity evolves and is maintained most readily in spatially or temporally heterogeneous conditions (Scheiner, 2013), but including these factors was beyond the scope of our study. Experiments in which the magnitude of environmental variability is directly manipulated will be key for future advances in this area.

We found evidence for adaptive plasticity in two root traits, underscoring the importance of measuring functional traits most directly impacted by soil selection pressures. We had predicted that the natural population would be more poorly adapted to anthropogenic stressors than the intense urban population. Our results supported this expectation for root mass ratio plasticity, because although plastic increases in root mass ratio were positively associated with fitness across all conditions and populations, the natural and intense urban populations differed in whether they primarily responded to stress via adaptive increases or maladaptive decreases in root mass ratio. Plants in stressful environments are thought to invest more in belowground biomass, as indicated by higher root:shoot ratios (Chapin et al., 1993). Soil environments are heterogenous, and root trait plasticity is one way in which plants exploit patches of nutrients (Hodge, 2004). If roots can plastically grow toward nutrient‐rich soil patches, then they might plastically grow away from a given soil stressor, therefore increasing belowground biomass allocation to escape stress. Studies on root trait plasticity in agricultural systems have shown that increased root trait plasticity is positively associated with drought resistance (Kano‐Nakata et al., 2011) and nutrient acquisition in phosphorous limited soil (Kumar et al., 2019). However, experimental support is lacking across other stressful conditions. Our results suggest that the ability to increase root mass ratio in response to stress has important fitness consequences and is conserved across contexts, highlighting the central importance of this root trait. The population‐specific variability in root mass ratio plasticity we observed is a novel finding that suggests populations exposed to a greater degree of anthropogenic stress may have evolved enhanced plasticity as a result.

Whether plasticity in specific root length was positively or negatively associated with fitness was apparently population dependent, with increasing specific root length in response to stress apparently adaptive for the natural and intense urban source populations but maladaptive for the rural and moderate urban source populations. For the latter populations, fitness benefits from maintaining shorter and thicker fine roots (low specific root length plasticity) under zinc stress make intuitive sense as thicker roots can act as barriers to metals in the soil (Sofo et al., 2017). However, plastically increasing specific root length via longer and thinner fine roots in response to zinc stress increases root surface area and should therefore increase susceptibility to zinc toxicity. Thus, the mechanisms underlying stress responses by plants from our natural and intense urban populations are not entirely clear. Phenotypic plasticity in response to stress can be beneficial for fitness in specific contexts (Chevin & Hoffmann, 2017), but further work is needed in this context to assess whether the patterns we found are generalizable and, if so, why.

### 4.3 Stress treatment effects

Although there is some evidence that reed canary grass has genetic (Haiminen et al., 2014; Maeda et al., 2006) and physiological (Polechońska & Klink, 2014) mechanisms that allow it to tolerate salt stress, our salt treatments were uniformly detrimental for traits related to productivity. This is not surprising, as excess salt reduces biomass production and growth because sodium ions in soil are taken up via potassium pathways, reducing potassium uptake (Czerniawska‐Kusza et al., 2004). In one 6‐week study on C4 grasses, symptoms from salt injury reduced total biomass by ≥70% when treated with 9.2 g Na/L solution (Hamilton et al., 2001). Concentrations in our study far exceeded that threshold (38.7 g Na/L), although they were consistent with levels recorded for some polluted urban wetland soils (Kim & Koretsky, 2013). The lack of source population differences in salt stress responses could be an indication that our treatment was so extreme that no variation in reed canary grass tolerance could be detected, regardless of population‐level differences.

The lack of population‐level differences in salt tolerance in our study could also reflect the lack of clear differences in sodium exposure across our gradient of source population wetlands. Predictions about salt exposure are not just dependent on land use in the surrounding area, because after de‐icing applications salt moves via soil and water in a largely site‐dependent manner. For example, in one study that examined salt movement through the soil in a moderately urban environment, sodium from de‐icing salt application rapidly leached through the soil column immediately adjacent to impervious surfaces at or exceeding de‐icing application rates, resulting in minimal exposure time for plants (Cunningham et al., 2008). In contrast, in a study examining sodium concentrations in wetlands from a rural watershed over time, sodium from de‐icing salts accumulated in wetlands and streams despite high leaching rates (Kelly et al., 2008). Given such site‐to‐site variability in sodium exposure and thus potential selective pressure, consistent salt tolerance responses based only on surrounding land use may be unlikely. We did not characterize actual salt inputs and movement dynamics in our source population wetlands; thus, a gradient of historic selection pressures for salt tolerance may not have occurred across our urbanization gradient.

Similar to salt stress, we found no evidence of variable tolerance to copper stress across our reed canary grass populations. Intraspecific variation in copper tolerance has been documented across its native range (Marchand et al., 2014), so in theory copper‐tolerant populations might occur in sites with sufficiently strong selection pressures. The lack of population differences in our study could reflect the small number of source populations we used and/or the absence of sites with high soil copper concentrations. The copper dose in our study is similar to that in work examining reed canary grass' performance in phytostabilization, where at the experiment's end investigators found 0.007–0.048 mg Cu/g root tissue (Korzeniowska & Stanis, 2011). Copper‐treated plants in our study had a mean of 0.018 mg Cu/g root tissue, with plants exhibiting similar reductions in biomass (Korzeniowska & Stanis, 2011).

None of our reed canary grass populations were tolerant to excess zinc in the soil, consistent with previous work on this species (Korzeniowska & Stanis, 2011; Korzeniowska & Stanislawska‐Glubiak, 2017; Matthews et al., 2005). Aboveground traits associated with PC1 scores (aboveground biomass, height, and chlorophyll content) were all reduced under zinc stress, causing separation between zinc and control treatments along PC1. These results are not surprising, as zinc can shift from being a required micronutrient to being toxic across a relatively small concentration gradient (Marschner, 2011). Physiologically, excess zinc is thought to inhibit photosynthesis by binding to ribulose‐1,5‐bisphosphate carboxylase oxygenase (RuBisCO) in place of magnesium, lowering the affinity of RuBisCO for carbon dioxide (Van Assche & Clijsters, 1986). Zinc toxicity also induces chlorosis and reduces fine root growth (Ruano et al., 1988; Sagardoy et al., 2009; Sofo et al., 2017). Previous work done by Korzeniowska and Stanis (2011) found a 59% reduction in reed canary grass biomass when treated with 800 mg Zn/kg soil. Similarly, we found a 59% reduction in biomass when treated with 410 mg Zn/7.6 L soil. Additionally, zinc stress in our plants reduced chlorophyll content and increased specific leaf area and root mass ratio (which reflected decreasing aboveground biomass while maintaining belowground biomass).

## 5 CONCLUSION

Our study suggests that selective pressures on key traits as well as on trait plasticities may lead to plant populations that are exposed to anthropogenic stressors evolving enhanced stress tolerance and greater plasticity, thus contributing to repeatable population‐level differentiation across human‐modified landscapes. Maintaining or increasing belowground biomass (i.e., plastic increases in root mass ratio) under stress was adaptive in all stress contexts, suggesting that selection under a range of stress regimes may favor plasticity in this trait. Our results emphasize the importance of further study regarding this and other root traits whenever variation in soil characteristics is thought to be important.

## CONFLICT OF INTEREST

None declared.

## AUTHOR CONTRIBUTIONS

**Leah M. Weston:** Data curation (lead); formal analysis (lead); investigation (equal); methodology (equal); project administration (equal); visualization (lead); writing–original draft (equal); writing–review and editing (equal). **Kali Mattingly:** Writing‐original draft (equal); writing–review and editing (equal). **Charles T. C. Day:** Data curation (supporting); investigation (equal); methodology (equal); project administration (equal); writing–original draft (supporting). **Stephen M. Hovick:** Conceptualization (lead); formal analysis (supporting); funding acquisition (lead); methodology (equal); writing–original draft (supporting); writing–review and editing (equal).

## Data Availability

The morphological trait dataset and R script are available at https://doi.org/10.5061/dryad.zgmsbccbh.

## APPENDIX A

**TABLE A1:** Pearson's correlation coefficients for all trait pairs

Traits	Aboveground biomass	Height	Chlorophyll content	Leaf dry matter content	Specific leaf area	Belowground biomass	Root mass ratio	Specific root length	Fine root diameter	Fine root length
Aboveground Biomass	‐									
Height	0.6621*	‐								
Chlorophyll Content	0.7561*	0.4970*	‐							
Leaf Dry Matter Content	−0.1305	0.1198	−0.2059	‐						
Specific Leaf Area	−0.1883	−0.3624*	−0.2497*	−0.6463*	‐					
Belowground Biomass	0.5646*	0.1352	0.3272*	−0.3307*	0.1530	‐				
Root Mass Ratio	−0.1923	−0.3930*	−0.2446*	−0.3153*	0.3401*	0.6485*	‐			
Specific Root Length	−0.3039*	−0.1304	−0.1995	−0.1503	0.2976*	−0.1689	−0.0247	‐		
Fine Root Diameter	0.0944	−0.0414	0.0485	0.1441	−0.2293*	0.0247	0.0321	−0.6994*	‐	
Fine Root Length	0.0508	0.0284	0.1052	0.1019	−0.0845	−0.0974	−0.1909	0.0992	−0.4786*	‐

* *p* < .05.

**TABLE A2:** Principal Component Analysis trait loadings (species scores) that show the degree to which each trait is correlated with individual principal components (PC1 through PC3)

Traits	PC1	PC2	PC3
Aboveground Biomass	−0.5067	0.2408	0.1505
Height	−0.4505	−0.0418	0.2152
Chlorophyll Content	−0.4568	0.1723	0.2041
Leaf Dry Matter Content	−0.1062	−0.4952	−0.1593
Specific Leaf Area	0.3356	0.3749	0.1194
Belowground Biomass	−0.1725	0.5467	−0.0335
Root Mass Ratio	0.2208	0.4521	−0.2250
Specific Root Length	0.3069	−0.0206	0.4702
Fine Root Diameter	−0.1863	−0.0098	−0.6245
Fine Root Length	−0.0169	−0.1445	0.4323

**TABLE A3:** Statistics from adaptive plasticity models depicted in Figure A1

Traits (Stress Treatment)	Source population		Trait plasticity		Trait value	
	*F*	*p*	*F*	*p*	*F*	*p*
Chlorophyll Content (Salt)	0.979	.441	4.347	.064	1.308	.279
Chlorophyll Content (Copper)	0.678	.585	0.029	.868	0.026	.875
Chlorophyll Content (Zinc)	1.258	.341	2.518	.144	9.861	.011
Leaf Dry Matter Content (Salt)	0.730	.557	0.516	.489	0.140	.716
Leaf Dry Matter Content (Copper)	0.714	.566	0.533	.482	0.269	.615
Leaf Dry Matter Content (Zinc)	0.642	.605	2.519	.144	0.568	.468
Specific Leaf Area (Salt)	0.727	.559	0.463	.512	0.130	.726
Specific Leaf Area (Copper)	0.701	.573	0.446	.519	0.081	.782
Specific Leaf Area (Zinc)	0.654	.598	4.580	.058	0.096	.763
Fine Root Diameter (Salt)	0.857	.495	0.907	.363	2.295	.161
Fine Root Diameter (Copper)	0.770	.537	0.040	.845	1.914	.197
Fine Root Diameter (Zinc)	0.631	.611	0.029	.868	1.012	.338
Fine Root Length (Salt)	0.746	.549	0.504	.494	0.477	.506
Fine Root Length (Copper)	0.689	.579	0.011	.919	0.269	.615
Fine Root Length (Zinc)	0.606	.626	3.356	.097	3.836	.079

Note

Models used source population and trait plasticity to predict total biomass in the indicated stressor treatment. The trait value itself was also included as a covariate. *N* = 20 in each case. Numbers in bold represent significance at *p* < .05.
